# Effects of traditional Chinese medicine Xin-Ji-Er-Kang formula on 2K1C hypertensive rats: role of oxidative stress and endothelial dysfunction

**DOI:** 10.1186/1472-6882-13-173

**Published:** 2013-07-13

**Authors:** Ting-ting Yu, Kun Guo, Han-chun Chen, Chao-zong Lan, Jian Wang, Ling-ling Huang, Xing-hui Wang, Zhen Zhang, Shan Gao

**Affiliations:** 1 Department of Pharmacology, Key Laboratory of Antiinflammatory and Immunopharmacology of Education Ministry, Basic Medical College, Anhui Medical University, Hefei 230032, China; 2 Department of Neurosurgery, Suzhou Kowloon Hospital, Shanghai Jiaotong University School of Medicine, Suzhou, Jiangsu 215021, China; 3 Department of Traditional Chinese medicine, College of Pharmacy, Anhui University of Chinese Medicine, Hefei 230038, China; 4 Department of General Surgery, the First Affiliated Hospital of Anhui Medical University, Hefei, Anhui 230022, China

**Keywords:** Oxidative stress, Endothelial dysfunction, Antioxidant, Renovascular, Hypertension, Cardiac remodeling

## Abstract

**Background:**

XinJiErKang (XJEK), a Chinese herbal formula, is identified as an effective preparation to treat coronary heart disease and myocarditis. The aim of the study is to investigate the anti-hypertensive effects of XJEK by oral administration and also to find out whether the drug has any role in oxidative stress and vascular endothelial function.

**Methods:**

Clipping of the renal artery resulted in gradual elevation of the systolic blood pressure (SBP) which reached a plateau after 4 weeks of surgery. Treatment of hypertensive rats (20 mmHg higher than basic systolic blood pressure) with XJEK (6, 12, 24 g/kg/day) and fosinopril (15 mg/kg/day) respectively by intragastric administration started 4 weeks after surgery and continued for 4 weeks. The sham-operated (Sh-Op) controls received drinking water. BP was monitored weekly using tail-cuff apparatus. At the end of 8 wk, left ventricular systolic pressure (LVSP), left ventricular end-diastolic pressure (LVEDP), rate of rise of left ventricular pressure (±dp/dtmax) were examined (PowerLab 8/30, AD Instruments, Australia). The myocardial hypertrophy index was expressed as heart weight/body weight (HW/BW), the histological changes were investigated by hematoxylin and eosin (HE) and Van Gieson (VG) stain. Endothelium-dependent relaxations due to acetylcholine were observed in isolated rat thoracic aortic ring preparation. Superoxide dismutase (SOD) activity, malondialdehyde (MDA) and nitric oxide (NO) content in serum, contents of hydroxyproline (Hyp) in the ventricular tissue were assayed by xanthin oxidase method, thiobarbituric acid (TBA) method, Griess method and alkaline hydrolysis method, respectively. Angiotensin II (Ang II) content in serum was detected by radioimmunoasssay method.

**Results:**

XJEK therapy potently improved cardiac function, inhibited myocardial hypertrophy, improved cardiac pathology change, decreased the myocardial cross-section area (CSA), collagen volume fraction (CVF) and perivascular circumferential collagen area (PVCA), reduced the content of Hyp in the left ventricular tissue, inhibited the decrease of SOD activity and increase of MDA, Ang II content in serum. Moreover, treatment with XJEK improved endothelial dysfunction (ED) manifested by promoting endothelial-dependent vasodilation of thoracic aortic rings and enhancing the NO activity in serum.

**Conclusions:**

These findings suggest that administration of XJEK possess protective effects against 2K1C induced hypertension and cardiac remodeling in rats, preserve NO activity and endothelial function.

## Background

Human hypertension is usually a slowly-developing disorder from middle to old age. Hypertension is not only manifested by an increased arterial pressure, it also involves the complex structural and functional alterations of its target organs. Long-term hypertension often results in left ventricular hypertrophy, which is considered to be a risk factor for coronary heart disease, congestive heart failure (CHF), ventricular arrhythmia, and sudden death [1], as well as structural alteration of the vascular wall, which is manifested by endothelial dysfunction (ED), extracellular matrix deposition and medial layer thickening because of hypertrophy/hyperplasia and migration of vascular smooth muscle cells (VSMCs). Epidemiological data revealed that hypertension is responsible for 54% of stroke, 47% of ischemic heart disease, and 25% of other cardiovascular diseases worldwide; a total of 6.7 million deaths throughout the world (≈13.5% of all) are ascribed to high blood pressure [2]. On the other hand, chronic kidney artery diseases, such as renal artery stenosis, generally lead to hypertension, and one of the kidney related animal models of hypertension is 2-kidney, 1-clip (2K1C) model in which one of the renal arteries is subjected to partial stenosis by clip placement. Kidney ischemia results in increase in plasma renin and angiotensin activity [3] which in turn leads to persistent increase in blood pressure. In experimental renovascular hypertension, increased oxidative stress (OS) plays an important part in the pathogenesis of renovascular hypertension and the enhancement of the oxidation-sensitive signaling pathway [4]. Previous studies have reported that angiotensin II (Ang II) stimulates the production of reactive oxygen species (ROS) such as superoxide through the activation of membrane-bound NADH or NADPH oxidase [5,6]. Recently, ED is known to play important roles in the pathogenesis and progressiveness of hypertensive heart disease [7]. One of the key factors in ED is the overproduction of ROS which participates in the development of hypertension, atherosclerosis, diabetes, cardiac hypertrophy, heart failure, ischemia-reperfusion injury, and stroke [8].

Xin-Ji-Er-Kang (XJEK) is a topical Chinese herbal medicine (CHM) compound made from fourteen herbal medicines such as Panax ginseng, Astragalus mongholicus Bunge, Ophiopogon japonicus, Polygonatum odoratum and so on. Both clinical study and basic research have exhibited the curative effect of XJEK on hypertension induced coronary heart disease, virus myocarditis and toxic myocarditis [9,10]. XJEK has also been shown to exert protective effects against isoproterenol-induced ventricular remodeling in mice, which may be related to its actions in reducing the oxidative stress and improving the antioxidant activity of the body [11]. Our preliminary experiment results also demonstrated that XJEK prevented heart, kidney, vascular remodeling and injury in 2K1C induced hypertension rats [12].

The aims of this research, therefore, are to reveal whether XJEK can prevent 2K1C-induced hypertension and cardiovascular remodeling (CR) and, if so, to determine the underlying mechanism, focusing on the involvement of OS and ED.

## Methods

### Preparation of XJEK extract

XJEK consists of fourteen medicinal compositions as shown in Table 1. All of these herbs were purchased from Hefei Company of Traditional Crude Drugs (Hefei, China), and carefully authenticated by Dr. He-ping Huang (AnHui College of Traditional Chinese Medicine, HeFei, China). Voucher specimens (numbers were listed in Table 1) were deposited at the Herbarium of Nanjing University of Traditional Chinese Medicine (Nanjing, China). After drying, these plant materials were mixed in proportion and were macerated for 1 h at room temperature with eight times (v/w) distilled water. Then the whole mixture was decocted twice for 1 h each time. The filtrates were mixed and condensed and then dried by vacuum-drier at 60°C. The yield of XJEK extract was 25.6% (w/w) according to the original herbs. The resulting powder, stored at −20°C, was diluted to the concentrations needed with distilled water and filtered before use.

**Table 1:** Recipe of XJEK formulation

**Components**	**Voucher specimens number**	**Part used**	**Rate (%)**
Panax ginseng C.A. Mey.	NJUTCM-20110530	Root	11.71
Polygonatum odoratum (Mill.) Druce	NJUTCM-20110531	Rhizome	7.03
Panax pseudoginseng var. notoginseng (Burkill) G. Hoo & C.L. Tseng	NJUTCM-20110532	Root	3.09
Allium macrostemon Bunge	NJUTCM-20110533	Ramulus	7.80
Angelica sinensis (Oliv.) Diels	NJUTCM-20110534	Root	7.80
Ophiopogon japonicus (Thunb.) Ker Gawl.	NJUTCM-20110535	Root	7.80
Schisandra chinensis (Turcz.) Baill.	NJUTCM-20110536	Fruit	3.93
Salvia miltiorrhiza f. alba C.Y. Wu & H.W. Li	NJUTCM-20110537	Root	7.80
Sophora flavescens Aiton	NJUTCM-20110538	Root	7.80
Glycyrrhiza acanthocarpa (Lindl.) J.M. Black	NJUTCM-20110539	Rhizome	7.80
Astragalus mongholicus Bunge	NJUTCM-20110540	Root	11.69
Epimedium acuminatum Franch.	NJUTCM-20110541	Aerial part	7.80
Trichosanthes obtusiloba C.Y. Wu	NJUTCM-20110542	Seed	7.80
Dryobalanops aromatica C.F. Gaertn.	NJUTCM-20110543	Resin	0.15

### Animals and treatment

Fifty-four male Wistar rats (200 ± 10 g) were purchased from the Laboratory Animal Center of Nanjing Medical University. All procedures were performed in accordance with the protocol outlined in the Guide for the Care and Use of Laboratory Animals published by the US National Institute of Health (NIH publication no. 85–23, revised 1996) and approved by the Committee on the Ethics of Animal Experiments of An’hui Medical University. The animals were housed under standardized conditions, 12 h dark–light cycle in solid bottomed polypropylene cages, and received commercial rat chow ad libitum.

2K1C hypertension was induced by subjecting the animals to right renal artery clamping using a 0.25 mm silver clip under pentobarbital anesthesia. The animals were randomly assigned to one of six groups. 1: Sham-operated (Sh-Op) rats underwent the same surgical procedure, except for the placement of the renal artery clip. 2: Experimentally induced hypertensive model group that underwent right renal artery clamping (2K1C) and received tap water; 3: XJEK low dose group: Rats underwent 2K1C and received XJEK at 6 g/kg/day; 4: XJEK middle dose group: Rats underwent 2K1C and received XJEK at 12 g/kg/day; 5: XJEK high dose group: Rats underwent 2K1C and received XJEK at 24 g/kg/day; 6: Fosinopril group: Rats underwent 2K1C and received fosinopril at 15 mg/kg/day. Treatment with XJEK was started 4 weeks after 2K1C hypertension was induced and maintained for an additional 4 weeks. Body-weight (BW) and tail systolic blood pressure (SBP) were assessed weekly throughout the experimental period.

### Measurement of systolic blood pressure

SBP was measured in all groups using the tail-cuff apparatus (ALC-NIBP, Shanghai Alcott Biotech CO., LTD.) at weekly intervals. Before the measurements, the rats were warmed for 30 min at 28°C in order to allow the detection of tail artery pulsations and to achieve a steady pulse level. SBP was obtained by averaging 10 measurements.

### Haemodynamics and cardiac remodeling index

At the end of eight weeks, all animals were anaesthetized with pentobarbital (45 mg/kg, intraperitoneal injection), the right carotid artery was cannulated with a polyethylene catheter connected to a Statham transducer and then the catheter was inserted along the right coronary artery into the left ventricle, and the signals were recorded on a PowerLab 8/30 (AD Instruments, Australia) and digitally sampled (1 kHz) on a personal computer using a chart software (version 5.3). The left ventricular systolic pressure (LVSP), left ventricular end-diastolic pressure (LVEDP) and rate of rise of left ventricular pressure (±dp/dtmax) were recorded.

Thereafter, blood samples were collected and centrifuged for 10 min at 3000 rpm, and all samples were stored at −80°C for further use. Then the animals were killed by exsanguinations, and the thoracic cavity was opened to expose the still beating heart. The hearts were rapidly removed, rinsed in ice-cold 0.9% NaCl solution, blotted and weighed. The heart–weight index (HW/BW) was calculated by dividing the heart weight by the body weight.

### Measurement of plasma Ang II levels

Blood from the abdominal aorta was collected into a chilled glass tube containing protease inhibitors and Enalapril to inhibit ex vivo conversion of Ang I to Ang II (Sigma, St. Louis, MO). After elution from the column with 90% methanol, samples were dried and reconstituted for radioimmunoassay (RIA). The RIA for Ang II was performed using ^125^ I-angiotensin II (Perkin-Elmer, Foster City, CA) and rabbit anti-Ang II antibody (Phoenix Pharmaceuticals, Inc., Belmont, CA) with cross-reactivity of <2% for Ang II precursors and degradation products. After incubation for 48 h at 4°C, bound and free Ang II was separated with dextran-coated charcoal. The supernatant was counted with a gamma-counter (ICN, Costa Mesa, CA). The ratio B/B_o_ was corrected for non-specific binding, expressed as a percentage of maximal binding, and read against a standard curve (log-logit transformation).

### Histological and morphological analyses of the heart and thoracic aorta

After weighing the heart, 70% of the upper hearts were frozen in liquid nitrogen for measuring the contents of hydroxyproline, and the other 30% were fixed in 10% formalin for histological analysis. Briefly, paraffin sections (5 μm) were cut and stained with hematoxylin and eosin (HE) and Van Gieson (VG). Thereafter, the myocyte cross-sectional area (CSA), perivscular collagen area (PVCA) and collagen volume fraction (CVF) were quantitatively analysed with NIH Image 1.61 software (National Institutes of Health Service Branch) in digitalized microscopic images as has been previously described [13].

Thoracic aortas were removed from rats and cleaned before use, and then tissues were cleaned and fixed in formaldehyde. Paraffin-embedded thoracic aorta (5 μm) was cut, dewaxed and stained with HE. The structural changes of aorta were investigated using a light microscope. Area of total aorta (TAA), area of lumen (LA), CSA, aorta radius (AR), luminal radius (L), and media thickness (M) of aorta were recorded under a light microscope, and the ratio of M/L was calculated as has been previously reported [13].

### Isolated vascular ring experiments

Isolated vascular ring experiments were performed as we previously described [14], with minor modification. In brief, four-millimeter ring segments of the descending thoracic aorta were dissected and mounted in individual organ chambers filled with Krebs buffer (composition in mM): NaCl 118, KCl 4.75, NaHCO_3_ 25, MgSO_4_ 1.2, CaCl_2_ 2, KH_2_PO_4_ 1.2, glucose 11. The solution was continuously gassed with a 95% O_2_ and 5% CO_2_ mixture and maintained at 37°C. Rings were stretched to 0.5 g of resting tension by means of two L-shaped stainless steel wires, which were inserted into the lumen and attached to the chamber and to an isometric force-displacement transducer. Rings were equilibrated for 60 to 90 min, during which, tissues were restretched and washed every 30 min with warm Krebs solution. The concentration-relaxation response curves to acetylcholine (10^-8^-10^-4^ mol/L) were performed in intact rings precontracted by 10^-4^ mol/L phenylephrine. Relaxant responses to acetylcholine were expressed as a percentage of precontract induced by phenylephrine.

### Measurement of nitric oxide (NO), total superoxide dismutase (SOD) activities, malondialdehyde (MDA) content in serum and hydroxyproline (Hyp) content in cardiac tissue

The methods of measuring NO have been described previously [15]. Because of its instability in physiological solutions, most of the NO was rapidly converted to nitrite (NO_2_^-^) and further to nitrate (NO_3_^-^). Serum levels of NO_2_^-^/NO_3_^-^ were measured using NO Detection Kit according to the manufacturer’s instruction. Briefly, nitrate was converted to nitrite with aspergillus nitrite reductase, and the total nitrite was measured with the Griess reagent. The absorbance was determined at 540 nm with a spectrophotometer.

As has been described previously [16], MDA content was measured using thiobarbituric acid reactive substances (TBARS) assay following the manufacturer’s instruction (Jiancheng Institute of Bioengineering Company, Nanjing, China) by measuring the absorbance value at wavelength of 532 nm. SOD activity was measured using xanthine oxidase method to measure the absorbance value at 550 nm with SOD kit (Jiancheng Bioengineering, Nanjing, China).

The contents of Hyp in cardiac muscle were measured as described formerly [13] according to the explanations provided by the manufacturer.

### Statistical analysis

All data are expressed as mean ± SD. For all the statistical tests, multiple comparisons were performed by one-way ANOVA with Tukey–Kramer exact probability test. The least-squares method was used for linear correlation between selected variables. Statistical significance was accepted at P < 0.05.

## Results

### Effect of XJEK on SBP

**Figure 1:**
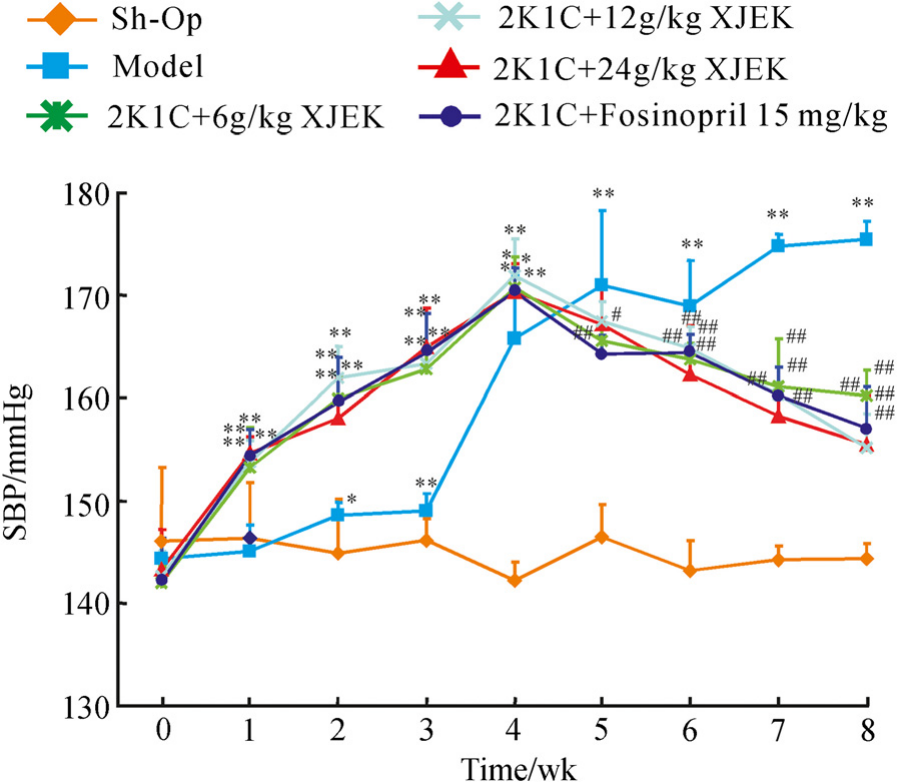
**Development of SBP in the six experimental groups during a 8-week period.** Nine time points of SBP were measured using tail-cuff apparatus measurement in each group. Data are expressed as the mean ± SD, n = 8 ~11. ^*^P < 0.05, ^**^P <0*.*01 *vs* Sh-Op group; ^#^*P* < 0.05, ^##^*P* < 0.01 *vs* model group.

SBP was considerably lower in the XJEK and fosinopril treated hypertensive rats as compared to experimentally induced hypertensive model group. A progressive reduction in BP was observed in XJEK and fosinopril treated groups from 5 week (P < 0.05, P < 0.01; Figure 1). At the end of 8 weeks, experiment animals treated with XJEK (each dose) and fosinopril demonstrated reduced SBP significantly, which was near to the SBP of Sh-Op group rats.

### Effect of XJEK on haemodynamic parameters

The measurements of *in vivo* left ventricular function for all groups were measured 8 weeks after 2K1C. As shown in Table 2, systolic cardiac parameters, including LVSP, LVEDP, +dp/dt_max_, and diastolic cardiac parameter -dp/dt_max_, were all significantly elevated in model group rats. These changes could also be prevented by treatment with XJEK in a dose-dependent manner. The same results were observed in positive drug-fosinopril treated group.

**Table 2:** **Effects of XJEK on cardiac function in 2K1C hypertensive rats (mean ± SD, *n*** **= 8 ~ 11)**

**Group**	**LVSP(mmHg)**	**LVEDP(mmHg)**	**+dP/dt**_**max**_**(mmHg/s)**	**-dP/dt**_**max**_**(mmHg/s)**
Sh-Op	113.07 ±11.66	27.56 ±9.11	7672.57 ± 2158.28	−7046.04 ±1501.92
Model	149.21 ± 20.68^**^	46.93 ± 19.92^**^	8477.46 ± 1123.74	−7483.98 ±1122.43
XJEK				
6 g/kg	121.34 ± 16.99^##^	40.88 ±7.40	7358.72 ± 1389.27	−7679.91 ±1906.72
12 g/kg	115.61 ± 14.16^##^	35.66 ±9.49	7140.11 ± 2493.83	−7666.94 ±1235.32
24 g/kg	113.60 ± 15.86^##^	37.93 ±4.28	6310.59 ± 1588.00^##^	−7015.20 ± 1162 .04
Fosinopril				
15 mg/kg	110.53 ± 11.26^##^	36.65 ±12.28	6514.32 ±2417.83^#^	−7152.12 ±1789.97

*LVSP* left ventricular systolic pressure, *LVEDP* left ventricular end-diastolic pressure, *+dp/dt*_*max*_ maximal rate of left ventricular systolic pressure, *-dp/dtmax* maximal rate of left ventricular diastolic pressure. ^**^P < 0.01 *vs* Sh-Op group; ^#^*P* < 0.05, ^##^*P* < 0.01 *vs* model group.

### Effect of XJEK on cardiac remodeling in 2K1C rats

**Figure 2:**
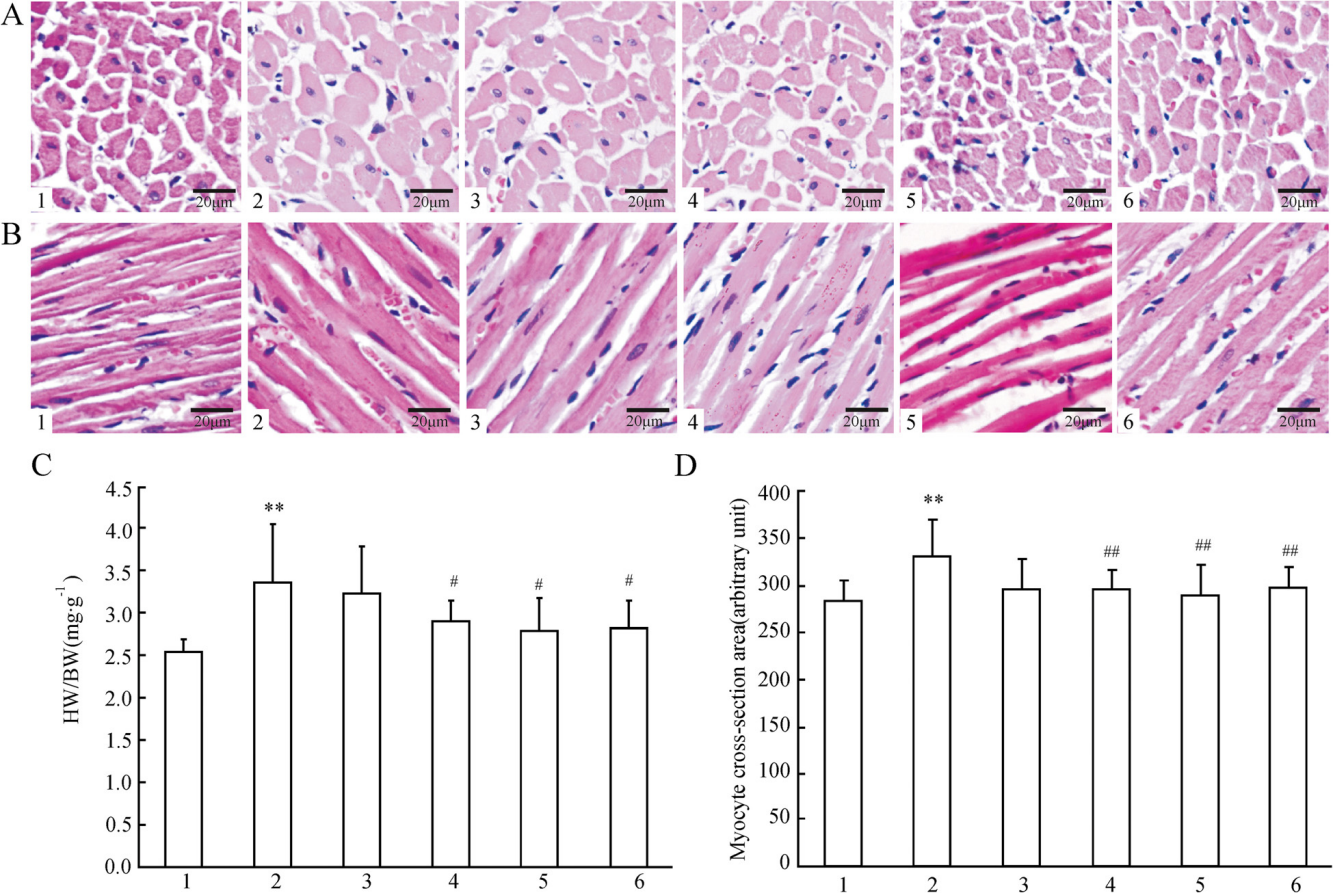
**Effects of XJEK on HW/BW, myocyte CSA of cardiac tissue in 2K1C rats. (A)** Representive figure of myocyte cross-section (HE staining, ×400); **(B)** Representive figure of myocyte long axis (HE staining, ×400); **(C)** Statistic results of HW/BW; **(D)** Statistic results of myocyte CSA. 1: Sh-Op group; 2: model group; 3:2K1C + XJEK 6 g/kg group; 4:2K1C + XJEK12g/kg group; 5: 2K1C + XJEK 24 g/kg group; 6:2K1C + fosinopril 15 mg/kg group. The HW/BW ratios, myocyte CSA increased significantly compared with the Sh-Op group. XJEK at all doses for 4 weeks could reverse these pathological changes, as well as the positive control fosinopril. Data are expressed as mean ± SD, *n* = 8 ~ 11. ^**^P <0*.*01 *vs* Sh-Op group; ^#^*P* < 0.05, ^##^*P* < 0.01 *vs* model group.

**Figure 3:**
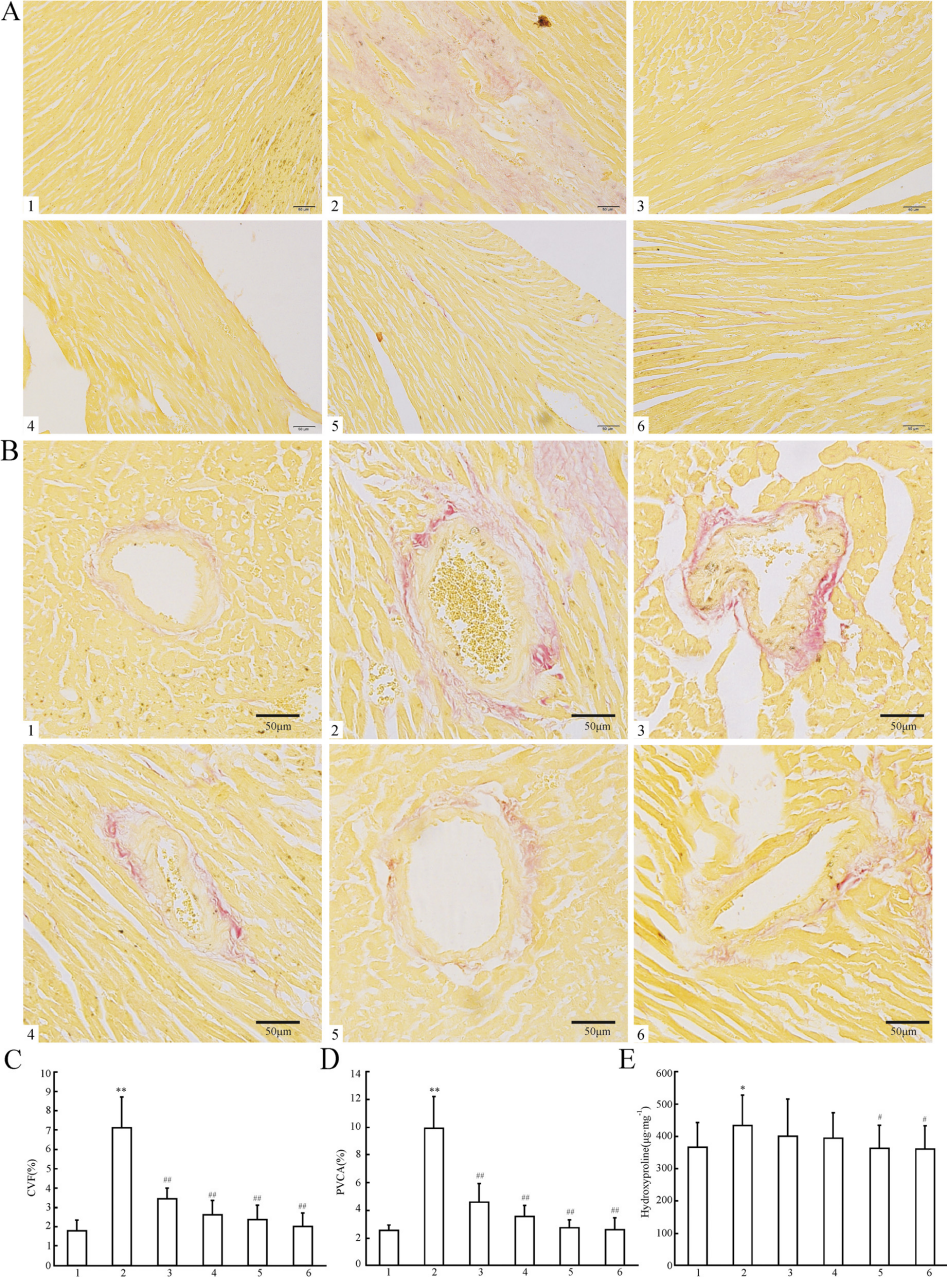
**Effects of XJEK on myocardial fibrosis, perivascular fibrosis, and cardiac Hyp content in 2K1C rats. (A)** Representive figure of myocardial fibrosis (VG staining, ×400); **(B)** Representive figure of perivascular fibrosis (VG staining, ×400); **(C)** Statistic results of myocardial fibrosis; **(D)** Statistic results of perivascular fibrosis; **(E)** Statistic results of cardiac Hyp content in cardiac tissue. Group divided ibidem. Levels of myocardial and perivascular fibrosis, and the cardiac Hyp content all increased significantly compared with the Sh-Op group. XJEK at all doses for 4 weeks could reverse these pathological changes, as well as the positive control fosinopril. Data are expressed as mean ± SD, *n* = 8 ~ 11. ^*^P < 0.05, ^**^P <0*.*01 *vs* Sh-Op group; ^#^*P* < 0.05, ^##^*P* < 0.01 *vs* model group.

Histology of the hearts from the experimentally induced hypertensive model group rats showed that myocyte CSA, and levels of CVF, PVCA increased significantly (^**^P < 0.01) as compared with those of the Sh-Op group (Figure 2-A,B,D and Figure 3-A,B,C,D). Morphological hypertrophy of heart was characterized by an increase in HW/BW ratios, which boosted significantly (^**^P < 0.01) compared with those of the Sh-Op group (Figure 2-C), whereas the BW showed no significant difference among the groups (data not shown). Moreover, the hydroxyproline content – reflecting the collagen level in cardiac tissue and the extent of myocardial fibrosis increased by 17.86% in experimentally induced hypertensive model group rats as compared with that of the Sh-Op group (Figure 3-E). XJEK at all doses for 4 weeks could reverse those pathological changes, as well as positive drug-fosinopril.

### Effect of XJEK on aortic remodeling in 2K1C rats

**Figure 4:**
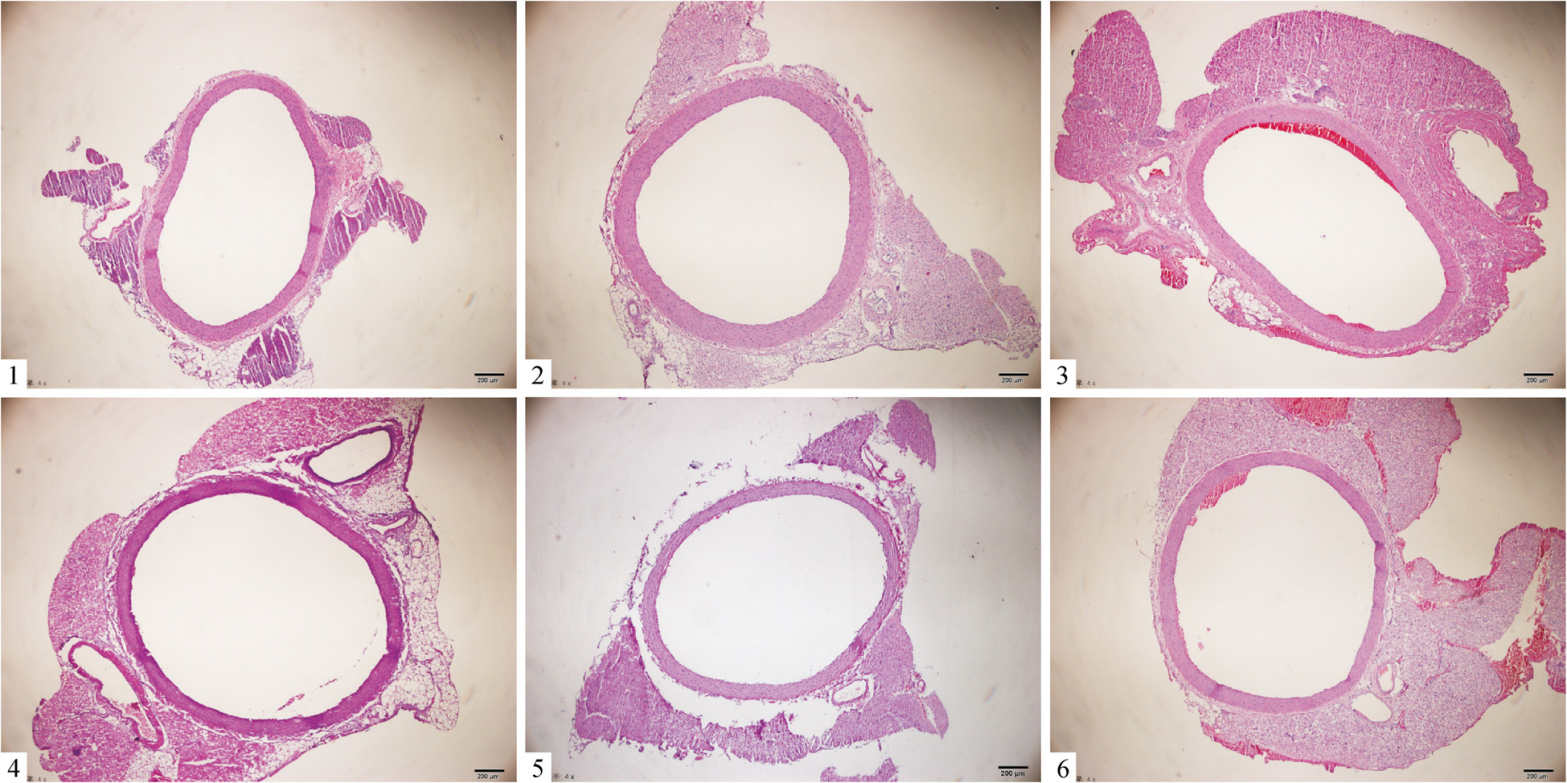
**Representative figure of aortic remodeling in different groups.** (HE staining, ×40) Group divided *ibidem*.

The vascular remodeling of the upper thoracic aorta exposed to 2K1C rats was observed at the end of 4th week. Compared with Sh-Op group rats, the values of the area of the TAA, LA, CSA, AR, M, and M/L ratio of the aorta in 2K1C rats were markedly increased. These changes could be prevented by treatment with XJEK at all doses for 4 weeks, as well as the positive drug (Table 3, Figure 4).

**Table 3:** **Effect of XJEKs on aorta remodeling in 2K1C rats (mean ± SD, *n*** **= 8 ~ 11)**

**Group**	**TAA (×10**^**3**^**um**^**2**^**)**	**LA (×10**^**3**^**um**^**2**^**)**	**CSA (×10**^**3**^**um**^**2**^**)**	**CSA/TAA (%)**	**AR (um)**	**Lumen (um)**	**Media (um)**	**Media/lumen (%)**
Sh-Op	2671.83 ± 108.18	2354.67 ± 99.27	317.17 ± 56.36	11.86 ± 1.99	374.58 ± 16.07	339.72 ± 18.89	34.86 ± 4.85	10.33 ± 1.90
Model	2819.57 ± 82.65^**^	2454.29 ± 71.59^*^	365.29 ± 86.36	12.92 ± 2.81	405.23 ± 13.43^**^	357.43 ± 12.14^*^	47.80 ± 5.27^**^	13.39 ± 1.52^**^
XJEK								
6 g/kg	2854.00 ± 162.03	2505.29 ± 176.62	348.71 ± 35.81	12.27 ± 1.66	408.76 ± 20.55	364.43 ± 24.35	44.32 ± 4.79	12.27 ± 2.10
12 g/kg	2764.00 ± 185.83	2411.33 ± 250.26	352.67 ± 66.40	12.90 ± 3.13	400.35 ± 26.12	354.46 ± 29.84	45.89 ± 4.95	13.06 ± 2.31
24 g/kg	2694.33 ± 82.08^##^	2379.67 ± 65.31^#^	314.67 ± 45.65^#^	11.66 ± 1.52	383.59 ± 11.80^##^	347.32 ± 16.20	36.27 ± 11.11^##^	10.54 ± 3.54^#^
Fosinopril								
15 mg/kg	2692.13 ± 77.46^##^	2410.13 ± 111.76	282.00 ± 56.86^##^	10.50 ± 2.28^#^	384.36 ± 26.74^#^	347.05 ± 33.86	37.30 ± 13.02^#^	11.11 ± 4.93

*TAA* area of total aorta, *LA* area of lumen, *CSA* cross-sectional area, *AR* aorta radius. The vascular remodeling of the upper thoracic aorta exposed to 2K1C was observed at the end of 4^th^ week, which could be prevented by treatment with XJEK at all doses for 4 weeks, as well as the positive drug fosinopril. ^*^P < 0.05, ^**^P <0.01 *vs* Sh-Op group; ^#^*P* < 0.05, ^##^*P* < 0.01 *vs* model group.

### Effect of XJEK on endothelial dysfunction in 2K1C rats

**Figure 5:**
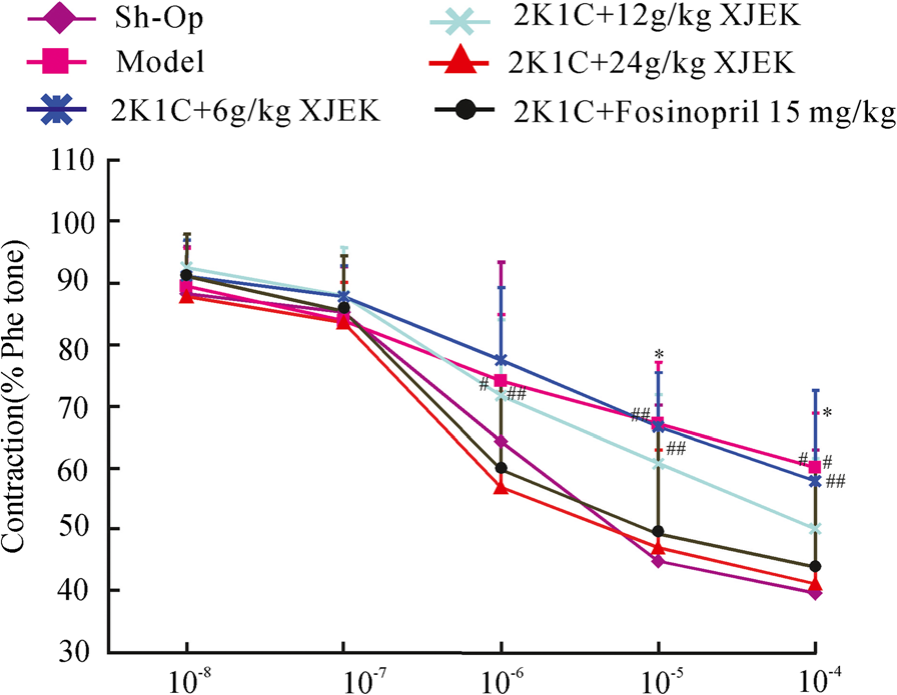
**XJEK and positive drug decrease contraction of the thoracic aorta from 2K1C rats.** Data are expressed as the mean ± SD, *n* = 8 ~11. ^*^P < 0.05 *vs* Sh-Op group; ^#^*P* < 0.05, ^##^*P* < 0.01 *vs* model group.

Aortic rings from unilateral renal clipping treated animals showed strongly reduced endothelium-dependent vasodilator responses to acetylcholine in arteries stimulated by phenylephrine compared to those in the Sh-Op (Figure 5). The aortic rings obtained from 2K1C rats treated with both XJEK and fosinopril showed a significant increase in vasodilatation induced by acetylcholine compared to the rings from the model group.

### Effect of XJEK on serum Ang II content

**Figure 6:**
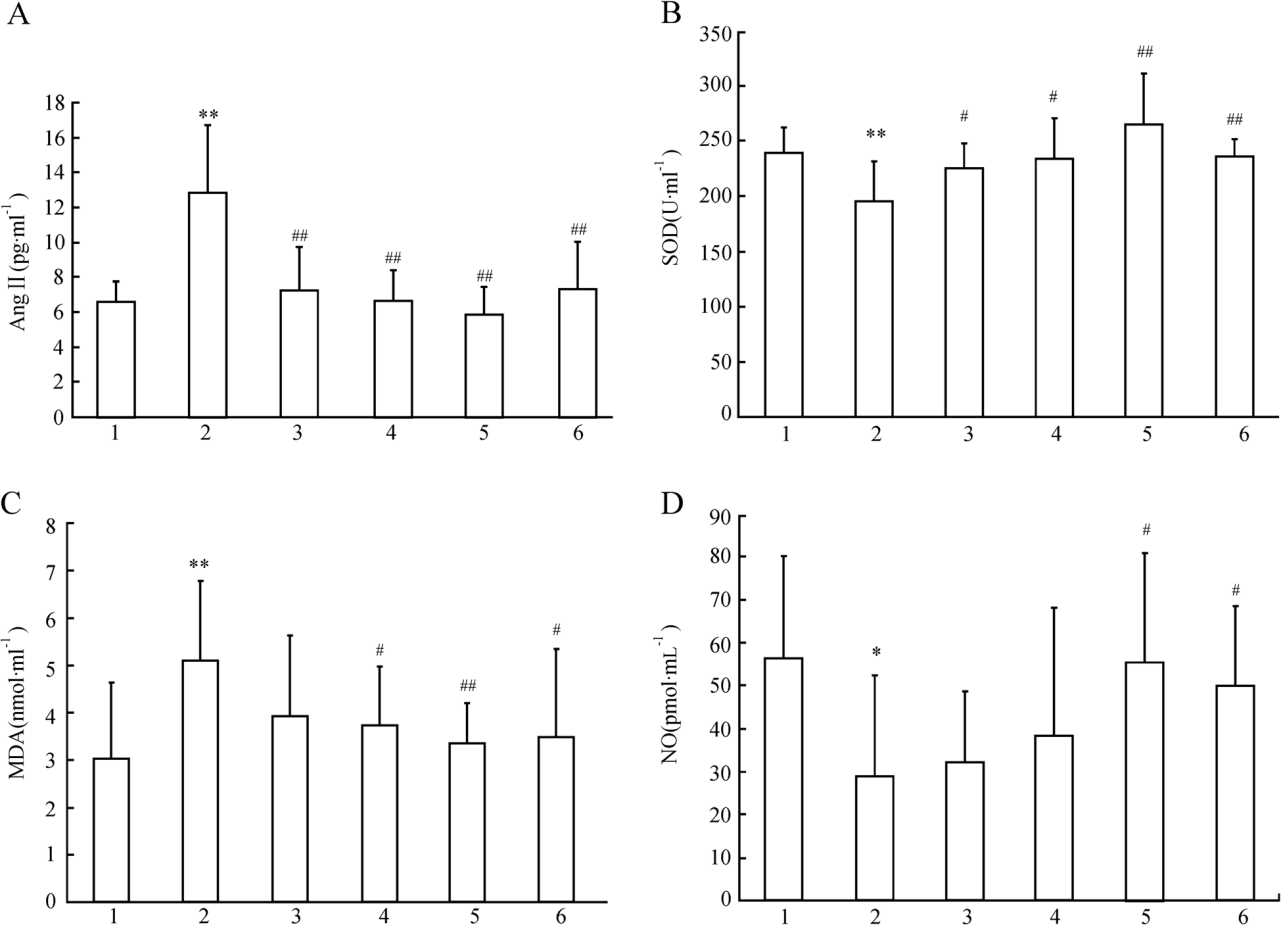
**Effects of XJEK on serum Ang II concentrations (A), SOD content (B), MDA content (C) and NO content (D) in 2K1C rats.** Group divided *ibidem.* Data are expressed as mean ± SD, *n* = 8 ~ 11. ^*^P < 0.05, ^**^P <0*.*01 *vs* Sh-Op group; ^#^*P* < 0.05, ^##^*P* < 0.01 *vs* model group.

The concentrations of Ang II measured in serum after 8 weeks are shown in Figure 6. Ang II content in 2K1C treatment is obviously increased than that in the Sh-Op group. Besides, administration of XJEK markedly reduced Ang II concentration in a dose-dependent manner, as well as the fosinopril group (P < 0.01, Figure 6-A).

### Effect of XJEK on serum SOD activity, MDA content and NO content in 2K1C rats

Lower SOD was found in 2K1C hypertensive rats compared with Sh-Op, and XJEK treatment restored SOD activity (Figure 6-B). Serum MDA was observably increased in the 2K1C group in 4th wk compared with Sh-Op group. Administration with XJEK at all doses for 4 weeks inhibited the increase of serum MDA markedly (Figure 6-C). Serum NO contents were significantly decreased in 2K1C group in comparison with Sh-Op group and XJEK administration at all doses for 4 weeks increased NO contents markedly (Figure 6-D).

## Discussion and conclusions

The main findings of the present study reveal that treatment with XJEK prevents hypertension and cardiovascular remodeling in unilateral renal clipping rats, which seems to be related to the attenuation of OS. Furthermore, the results also indicate that XJEK moderate ED, confirmed by increasing serum NO production, which may cooperate with their beneficial cardiovascular effects. This is the first study to compare the effects of significantly different doses of XJEK on biochemical, morphological and functional alterations caused by 2K1C hypertension.

Renovascular hypertension in the 2K1C model is characterised by elevated Ang II expression resulted from ischemia in the clipped kidney and shear stress in the non-clipped kidney, sustained increase of blood pressure and the following cardiovascular remodeling. CR is defined as genome expression, especially the re-expression of fetal isoforms such as atrial natriuretic peptide (ANP) [17], cellular, including the enlargement of cell size and mass of individual cardiomyocytes without an increase in cell number; and interstitial fibrosis, manifested clinically as changes in the size, shape, and function of the heart after cardiac injury [18]. Various harmful sequelae of cardiovascular diseases and conditions such as coronary heart disease, stroke, congestive heart failure and sudden death are known to be aggravated by CR [19,20]. Apart from CR, this experimental model is also associated with changes in the structural and mechanical properties of the arteries (named vascular remodeling, VR), including arterial wall hypertrophy and an increase in media/lumen ratio, changes in vascular wall stiffness ascribing to the up-regulation of matrix metalloproteinases (MMPs) [21] and severe renal histopathological lesions (such as glomerular hypertrophy, tubulointerstitial damage and glomerular volume) [22]. These changes increase vascular resistance to flow, further compounding the elevation in blood pressure. In accordance with these previous studies, the present study reveals that 2K1C treatment results in prominent cardiovascular remodeling, manifested as elevation in HW/BW and CSA, and increase in collagen deposition, wall thickness, TAA, media thickness. Our present results also demonstrate that chronic oral administration with XJEK prevents hypertension and cardiovascular remodeling in this 2K1C-induced hypertensive rat model.

Ang II is the primary effector molecule of the renin–angiotensin system. It is primarily recognized for its role in the regulation of arterial pressure and blood volume [23]. In addition to its pressor effect, Ang II has a variety of non-hemodynamic actions. For example, Ang II–induced cell growth and fibrosis may bring about left ventricular hypertrophy and vascular remodeling. Recently, the role of OS in cardiovascular diseases has been characterized. Many of the deleterious cellular phenotypes presented in hypertrophied and failing myocardium might contribute to ROS and OS, and it is clear that NADPH oxidase-derived ROS production plays a critical role in the hypertension induced by Ang II. In a model of in vivo cardiac hypertrophy induced by short-term (7–14 day) sub-pressor infusion of Ang II [24], it has been found that increase in heart/body weight ratio, myocyte area and mRNA expression of ANF and β-MHC, whilst administration of a NADPH oxidase inhibitor reduces vascular O_2_^.-^ production and attenuates Ang II-induced increase in blood pressure [25]. This NADPH oxidase-derived ROS function as secondary messengers activating myriad redox-sensitive downstream targets, such as RAS, c-src, the MAPKs, the PI3 kinase (PI3K)/Akt pathway, NF-κB, AP-1, HIF-1 and others, the significant role of which has been confirmed in cardiovascular remodeling [26]. Furthermore, these excessively generated ROS may directly react with NO, thereby stimulating the production of the NO/superoxide anion reaction product peroxynitrite (ONOO^-^), accordingly, increased ONOO^-^ may impair the function of the endothelial NO synthase (eNOS) by reducing the bioavailability of its co-enzyme tetrahydrobiopterin (BH_4_), which is required for NOS dimer formation and only if in this coupled state eNOS consumes NADPH and generates NO and *L*-citrulline from *L*-arginine and O_2_. The decreasing bioavailability of BH_4_ induces an unstable structure of eNOS, a phenomenon known as ‘eNOS uncoupling’ and on protein gels, it appears more as a monomer, and electrons become diverted to molecular oxygen rather than to *L*-arginine, resulting in O_2_^-^ formation, which thereby causes an anti-cardiovascular remodeling NO-producing enzyme to become a ROS-producing one, and accelerates the cardiovascular remodeling process hence [27]. Our present study reveals that blunted endothelium-dependent relaxation response to acetylcholine in noradrenaline precontracted aortic rings and decrease NO content in serum, accompanied with OS in this 2K1C rats hypertensive rat model. *Ginseng, Astragalus mongholicus, Radix Ophiopogo-nis* and *Polygonatum odoratum* are the main components of XJEK formula. It has been reported that ginsenoside exists in *ginseng,* and total flavonoids of *Astragalus* (the active component of *Astragalus* mongholicus Bunge) and the decoction of *Radix Ophiopogonis* exhibit a potent antioxidant activity [28-30]. Our present study demonstrates that chronic treatment with XJEK prevents ED and OS in a doses-dependent mode. In conclusion, marked OS exists in the 2K1C hypertensive rat model as described in the data, which participates in cardiovascular remodeling and endothelial dysfunction, at least in part. Chronic treatment with XJEK prevents these pathological changes as well as the positive drug-fosinopril. The protective effect is most likely due to the ability of XJEK to attenuate OS and/or by improving ED and increasing NO release in this 2K1C hypertensive rat model.

## Competing interests

The authors have no actual or potential conflict of interest associated with this work.

## Authors’ contributions

GS, YTT and CHC participated in the design of the study data analyses and manuscript preparation. YTT, GK, LCZ and WXH conducted the assays and analyses. All authors read and approved the final manuscript.

## Pre-publication history

The pre-publication history for this paper can be accessed here:

http://www.biomedcentral.com/1472-6882/13/173/prepub
